# The Twentieth Century

**Published:** 1901-01

**Authors:** 


A Monthly Review of Medicine and Surgery.

*EDITOR:*

WILLIAM WARREN POTTER, M. D.

All communications, whether of a literary or business nature, books for review and exchanges should be addressed to the editor: 284 FRANKLIN STREET, BUFFALO, N. Y.

VOL. XL.—LVI. JANUARY, 1901. No. 6.

### THE TWENTIETH CENTURY.

WHATEVER differences of opinion may have existed last year with reference to the period when the nineteenth century should end, there can be no dispute over the fact that now the twentieth century has started out on its momentous career.

The century just closed has been remarkable for the advances in the arts and sciences, in which medicine has come in for its full share. It would be interesting to contrast the status of medicine a hundred years ago with that of today, but it would take a long chapter, and one that perhaps would weary our readers.

It is pleasant to contemplate that no profession, no calling, no guild, has done more to advance civilisation in the last hundred years, or, for that matter, during any of the centuries, than medicine. A former editor of this JOURNAL in writing of the status of the profession in 1855,[1] said:

> No institution on earth presents such a catholic uniformity of sentiment, or is so widely scattered in every land. Medical men from every town where civilisation exists, throughout the wide world, might meet in congress with common interests, a common code of ethics, and a common literature. Such a congress would represent all the various interests of learning; for it is a fact of glorious significance, that natural science and the useful arts owe half of their vigor to the discursive researches of medical men. A large proportion, probably more than half the common stock of human learning is found within the pale of our profession.

1. Dr. S. B. Hunt, Vol. X. (old series) p. 579.

If this were true nearly half a century ago, and none has challenged the truth of the statement, it is equally true today. Nay, even more; for in the last third of a century the relative proportions of the learning of the medical profession to the common stock of human knowledge has materially increased. Human life, too, has increased its longevity during the last generation or two under the fostering, tender care of the medical profession, which is ever busy with the problems of sanitary science, public and private hygiène, and everything pertaining to preventive medicine.

The dawning of the twentieth century witnesses the most triumphant era in the history of medicine. It would be almost too much to expect, or to predict, that as much in the way of advance will be accomplished as during the century just ended. But there is plenty of encouragement to the lover of work. The lines are to be strengthened, the front extended, the recruits drilled, and the general field of medicine is to be rendered invulnerable to attack.

It is possible, however, that our greatest danger is not from pressure from without, but from restlessness and discontent from within our own ranks. The desire for notoriety that soon seizes hold of some of our younger men, and the ambitions that overwhelm some of the older ones are to be carefully watched and curbed lest the ægis of our safety becomes imperiled.

One of the great evils of the period is, in our view, the tendency to multiply medical societies. The great number of these, local, state and national, become a great money tax in dues upon the younger physicians, not to mention the time absorbed in attending upon their meetings, and the cost of travel and maintenance pertaining to the same. It would appear to be the province of the better element in the profession to discountenance the increase of medical societies and to foster the reduction in number of those now existing. If the medical societies will amalgamate, consolidate, or dissolve, so as to reduce their number, they will, surely, in this way expand and increase their influence. The man who proposes the organisation of a new medical society is not only asking for something that is wholly unnecessary, but he is very near being a menace to the best interests of the guild of medicine.

We have not intended to discourse at length, or in detail on this or any other question of importance relating to the twentieth century of medicine, but merely to hint at a feature or two of moment.

